# The Sputa in Phthisis

**Published:** 1858-02

**Authors:** 


*The Sputa in Phthisis.*—Dr. J. L. C. Schrœder Van der Kolk, Professor in the University of Utrecht, claims to have discovered a certain sign by which the sputa of a phthisical patient may be distinguished from those of chronic catarrhal affections of the lungs. He regards the presence of *elastic pulmonary fibres* in the sputa as a certain sign of the existence of a vomica. —*Ibid.*

